# Effective adsorbent for the removal of methylene blue using natural serpentine/magnetite nanocomposites: Isotherm and kinetic study

**DOI:** 10.1016/j.heliyon.2024.e41063

**Published:** 2024-12-09

**Authors:** Amtul Basit, Zahida Yaqoob, Aliya Zahid, Shaista Ali, Beenish Shoukat, Abdul Khaliq, Muhammad Tajammal Chughtai, Rahila Batul, Muhammad Atiq Ur Rehman, Syed Wilayat Husain

**Affiliations:** a Department of Materials Science and Engineering, Institute of Space Technology, Islamabad, 44000, Pakistan; b Department of Chemistry, Government College University, Lahore, 54000, Pakistan; c College of Engineering, University of Hail, Saudi Arabia; d College of Pharmacy, University of Hail, Saudi Arabia

**Keywords:** Serpentines, Magnetite, Nanocomposites, Removal, Co-precipitation, Dye adsorption

## Abstract

The natural serpentine from Skardu, Pakistan, has been incorporated with magnetite (Fe_3_O_4_) to form a nanocomposite using a chemical co-precipitation method for the removal of methylene blue (MB) dye. Various techniques such as Field Emission Scanning Electron Microscopy (FESEM), Energy Dispersive X-ray spectroscopy (EDX), Fourier Transform Infrared spectroscopy (FTIR), Brunauer-Emmett-Teller (BET), and Vibrating Sample Magnetometer (VSM) have been employed for the characterization. It was found that the composite exhibits sheet-like morphologies, Mg- and Si-rich surfaces, a surface area of 20.8 m^2^/g, and magnetic properties. The adsorption study showed that the Freundlich isotherm and pseudo-second-order kinetics best described the process with the nanocomposite achieving a 98 % MB removal efficiency, outperforming the pure serpentine. These results highlight the potential of serpentine/Fe_3_O_4_ nanocomposite as an effective, cost-efficient adsorbent for wastewater treatment applications.

## Graphical abstract

**Figure:**
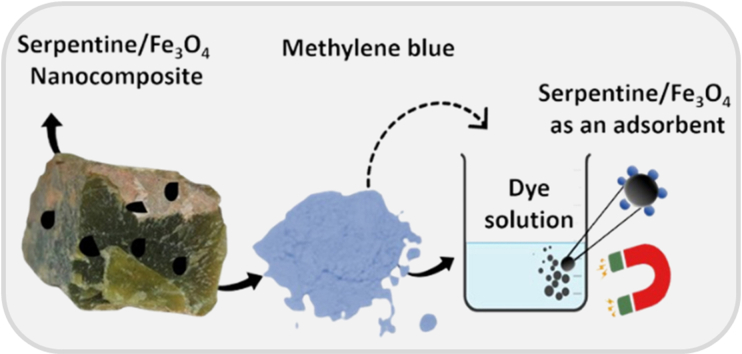

## 1 Introduction

The growth of the dyeing industry is becoming more extensive because it can provide materials such as leather and beautiful fiber colors. This huge production of the dyeing and printing industry leads to a large amount of wastewater [1]. Methylene blue (MB) is a common dye that is used for dyeing leather; this water-soluble dye is used for printing on cotton [2]. The major environmental concern is to remove MB from industrial effluents [3]. There are numerous methods to treat dye wastewater such as ion exchange, membrane method, flocculation sedimentation, chemical oxidation, and adsorption [4,5,6]. Adsorption is the most common and cost-effective method among others, which removes wastes from water due to high surface area resulting in large adsorption capacity [7]. Adsorption of different contaminants in water such as dyes, anions, heavy metals, and drug residue using cost-effective materials is often a promising approach for a sustainable future. Various materials have been studied to develop novel adsorbents for the treatment of wastewater. Zeolites, graphene nano-sheets, and clay minerals are among the synthetic and natural materials that have drawn interest due to their abundance, low cost, chemical inactivity, and superior adsorptive characteristics [[8], [9], [10], [11]]. However, different possible conventional and non-traditional forms of these effective adsorbents and cost-effective nanoparticles have already been applied to wastewater treatment in order to remove particular organic and inorganic contaminants [12,13]. Recently, magnetic adsorbents have become interesting for researchers because they can easily be recovered by using an external magnetic field [14]. Farahat et al. reported the magnetic particle with serpentine as an adsorbent for Cr (VI) removal from tannery wastewater [15]. Similarly, Nayl et al. prepared and characterized the magnetite talc nanocomposite as an effective adsorbent for Cr (VI) and organic dye [16]. Kalantari et al. developed Fe_3_O_4_/talc nanocomposite and used it to isolate heavy metals like Cu (II), Pb (II), and Ni (II) from water [17].

Serpentine minerals are phyllosilicates that are typically generated during the hydration of basic to ultrabasic rocks with a vast metamorphic stability field. Serpentine, Mg_3_Si_2_O_5_(OH)_4_ is widely abundant in Skardu that is mainly composed of one or more of the three magnesium silicate minerals [18,19]. The brucite octahedral magnesium hydroxide is held together by ionic and covalent bonds between tetrahedral silica sheets [8]. Serpentine is distinguished by the presence of numerous hydroxyl (OH) functional groups, along with water molecules that are attached to its surface [20]. Serpentine is frequently used as a decorative stone and as a raw material to produce asbestos and magnesium oxide with high purity [21]. Magnetic nanoparticles are considered the best adsorbents to eliminate all kinds of contaminants from water and wastewater [22,23]. Moreover, magnetite (Fe_3_O_4_) is simple to prepare and functionalize in the lab, it has been thoroughly investigated as a core material. Fe_3_O_4_ may be able to satisfy the rising need for biological applications, such as drug delivery, thermal treatment, and diagnostic magnetic resonance imaging due to its superparamagnetism and low toxicity [24,25]. Fe_3_O_4_ nanoparticles have shown to be highly effective in adsorbing heavy metal ions for removal. The adsorption process on magnetite occurs through a combination of electrostatic attraction and ligand exchange mechanisms [17].

To our knowledge, no work has been reported regarding the use of naturally abundant serpentine from Skardu for dye removal from waste water. In the present work, naturally abundant serpentine from Skardu has been incorporated with Fe_3_O_4_ nanoparticles for the removal of methylene blue (MB) from contaminated aqueous solutions. Both serpentine and its nanocomposite were characterized using FE-SEM, FTIR, VSM, and BET. Kinetic and isotherm models were investigated to determine the adsorption capacity. Langmuir and Freundlich models were analyzed to fit the adsorption data of serpentine and the serpentine/Fe_3_O_4_ nanocomposite for the removal of MB.

## 2 Materials and method

The following materials were required to produce the serpentine/Fe_3_O_4_ nanocomposite; ferrous chloride tetrahydrate (FeCl_2_.4H_2_O) (Fe (II)), sodium hydroxide (NaOH), and ferric chloride hexahydrate (FeCl_3_.6H_2_O) (Fe (III)), methylene blue (C_16_H_18_CIN_3_S, 98.5 % purity), and Ethanol (99.9 %) which were all purchased from Sigma Aldrich. Serpentine was collected from Shiger, Skardu. All these aqueous solutions were used with de-ionized water.

### 2.1 Synthesis of serpentine/Fe_3_O_4_ nanocomposite

**Fig. 1:**
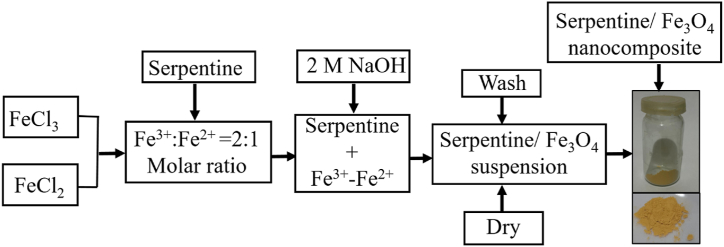
Experimental procedure for synthesis of serpentine/Fe_3_O_4_ nanocomposites.

For the synthesis of serpentine/Fe_3_O_4_ nanocomposites, the procedure reported in the literature was followed [26]. Firstly, 2.0 g of serpentine was mixed with 20 mL of deionized water and stirred for 1 h at 50 °C. In another beaker, 6.5 g of FeCl_3_.6H_2_O and 2.5 g of FeCl_2_.4H_2_O were dissolved in 40 mL of deionized water at room temperature with continuous stirring. The molar ratio of FeCl_3_ to FeCl_2_ was adjusted to 2:1 by adding specific amounts of Fe^3+^ and Fe^2+^. The contents of the two beakers were then mixed together, and the mixture was stirred for 1 h to allow the ions to adhere to the surface of the serpentine, resulting in a serpentine/Fe^3+^-Fe^2+^composites. Subsequently, 15 mL of freshly prepared NaOH (2.0 M) was added to the composite suspension with continued stirring. The resulting suspensions were centrifuged at 1000 rpm for 10 min, washed twice with ethanol and double-distilled water until a neutral pH was achieved, and then dried in an oven at 100 °C. The procedures were conducted at room temperature in a nitrogen gas environment to maintain an oxygen-free atmosphere. The experimental protocols are illustrated in Fig. 1.

### 2.2 Adsorption experiment

A stock solution of MB was prepared and diluted to the required concentration of 100 ppm (dye = 50 mg, distilled water = 500 mL), and a certain weight of adsorbent was employed in a 100 mL dye solution for both samples. The adsorbent was separated magnetically by using magnet and the analysis was done under the UV–visible spectroscopy for MB removal.

Equation (1) determined the adsorbed amount (q_t_) of MB using the formula:

Eq.1 $$q_{t}=V\frac{\left(C_{t-}C_{i}\right)}{W}$$

where C_i_ is the initial concentration (mg/L) of MB, W is the composite mass (g) C_t_ is the concentration of (mg/L) of dye after time (t), and V is the dye solution volume (L).

By adjusting the adsorbent dose (1–5 mg), time interval (10–60 min), and dye concentration (10–50 ppm), the adsorption of MB on serpentine and serpentine/Fe_3_O_4_ nanocomposites, produced by co-precipitation technique, were investigated. The dye concentration was adjusted from 10 to 50 ppm and the dye-adsorbent interaction time was measured. The mixtures were shaken at 125 rpm for 60 min using an orbital shaker (Techno ISO-207). 3 mg of produced serpentine/Fe_3_O_4_ nanocomposites and 20 ppm of metal ion solution were combined, and then the trial was carried out at room temperature for the investigation of the time factor. The adsorption of the MB gradually increases over time up to 60 min which further increase in agitation time may leads towards the greater removal.The adsorbent dose effect was also studied by keeping all the other factors constant (20 ppm dye concentration, time = 60 min). To ensure reproducibility, each kinetic experiment was repeated three times under identical conditions. The mean values are presented with standard deviations as error bars in the figures. The adsorption comparison between the serpentine/Fe_3_O_4_ nanocomposites and serpentine leads to conclude the better adsorption efficiency of serpentine/Fe_3_O_4_ nanocomposites.

### 2.3 Characterization

Field emission scanning electron microscopy (FESEM-EDS: Tescan-MIRA III) with an energy dispersive X-ray analyzer (EDX) was used to evaluate the morphology of the sample. To confirm the crystallinity of serpentine and serpentine/Fe_3_O_4_ nanocomposites, powdered samples were subjected to X-ray diffraction (XRD: Panalytical-PW3719) using Cu Kα radiations in the 2θ range from 20 to 80° (step size: 0.02). Fourier transform infrared spectroscopy (FTIR: Nicolet Summit LITE) in a transmittance mode was used to identify the functional groups in each sample. The scan speed was 4 m/s, and the range covered was 4000–400 cm^−1^. The magnetization behavior of the samples was evaluated using a vibrating sample magnetometer (LAKESHORE, 7407) to measure the magnetization at ambient temperature as a function of magnetic field strength (Oe). The surface area and pore size range for the serpentine and serpentine/Fe_3_O_4_ were computed using the Brunauer-Emmett-Teller (BET) and Barrett-Joyner-Halenda (BJH) models (V-Sorb 2800, GOLD APP Instruments), respectively. Nitrogen (N_2_) adsorption-desorption isotherm was obtained at liquid N_2_ temperature (77 K).

## 3 Results and discussion

### 3.1 SEM analysis

**Fig. 2:**
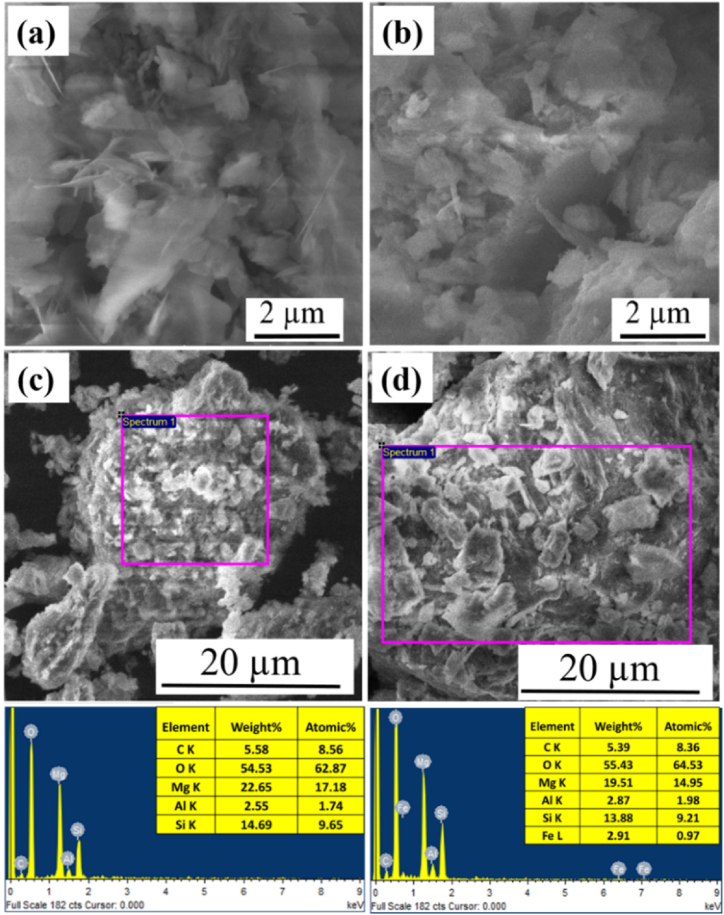
FESEM images (a) Serpentine (b) Serpentine/Fe_3_O_4_ along with EDX analysis showing elemental composition for (c) Serpentine (d) Serpentine/Fe_3_O_4_.

The surface morphologies of serpentine and serpentine/Fe_3_O_4_ were evaluated via FE-SEM and their chemical compositions were analyzed through EDX as illustrated in Fig. 2. The layered surface with a few big flakes; is usually similar to the talc structure (Fig. 2(a)) [27]. However, no significant morphological differences between serpentine and serpentine/Fe_3_O_4_ nanocomposite were observed as shown in (Fig. 2 b). Because serpentine has a high concentration of Mg, Si, and Al, the EDS analysis of the gray-colored patches reflects the chemical composition of serpentine (Fig. 2(c)). Similarly, EDS analysis of serpentine/Fe_3_O_4_ shows Fe, Mg, Si, and Al (Fig. 2(d)).

### 3.2 XRD analysis

**Fig. 3:**
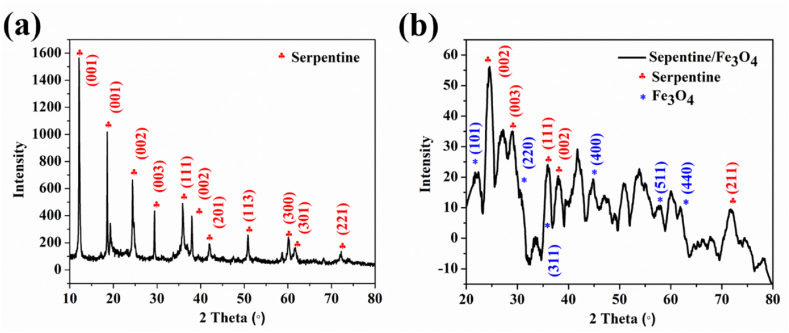
XRD pattern of (a) serpentine and (b) serpentine/Fe_3_O_4_ nanocomposite which matches the standard JCPDS cards.

The XRD patterns of serpentine and serpentine/Fe_3_O_4_ nanocomposite are shown in Fig. 3(a) and (b). The XRD pattern of serpentine and antigorite (JCPDS card No. 02-0095) exhibits a strong correlation with the minimal existence of talc mineral (JCPDS card no. 73-0147) at 2θ = 12.19ᵒ, 18.48ᵒ, 24.76ᵒ, 29.07ᵒ, 35.91ᵒ, 37.94ᵒ, 38.20ᵒ, 41.92ᵒ, 50.97ᵒ, 60.16ᵒ, 60.70ᵒ, 71.99ᵒ, and 72.19ᵒ which is shown in Fig. 3 (a) [15]. The distinctive peaks of magnetite (JCPDS card no. 89-0688) can be identified at 2θ = 22.12ᵒ, 30.69ᵒ, 36.03ᵒ, 44.90ᵒ, 58.14ᵒ and 62.04ᵒ (Fig. 3 b) [28]. All mineral phases were found in serpentine, according to the mineralogical data for the produced serpentine/Fe_3_O_4_ nanocomposite. The analysis of serpentine composition revealed that MgO and SiO_2_ are its primary constituents; these results also indicated from the EDX analysis.

### 3.3 FTIR analysis

**Fig. 4:**
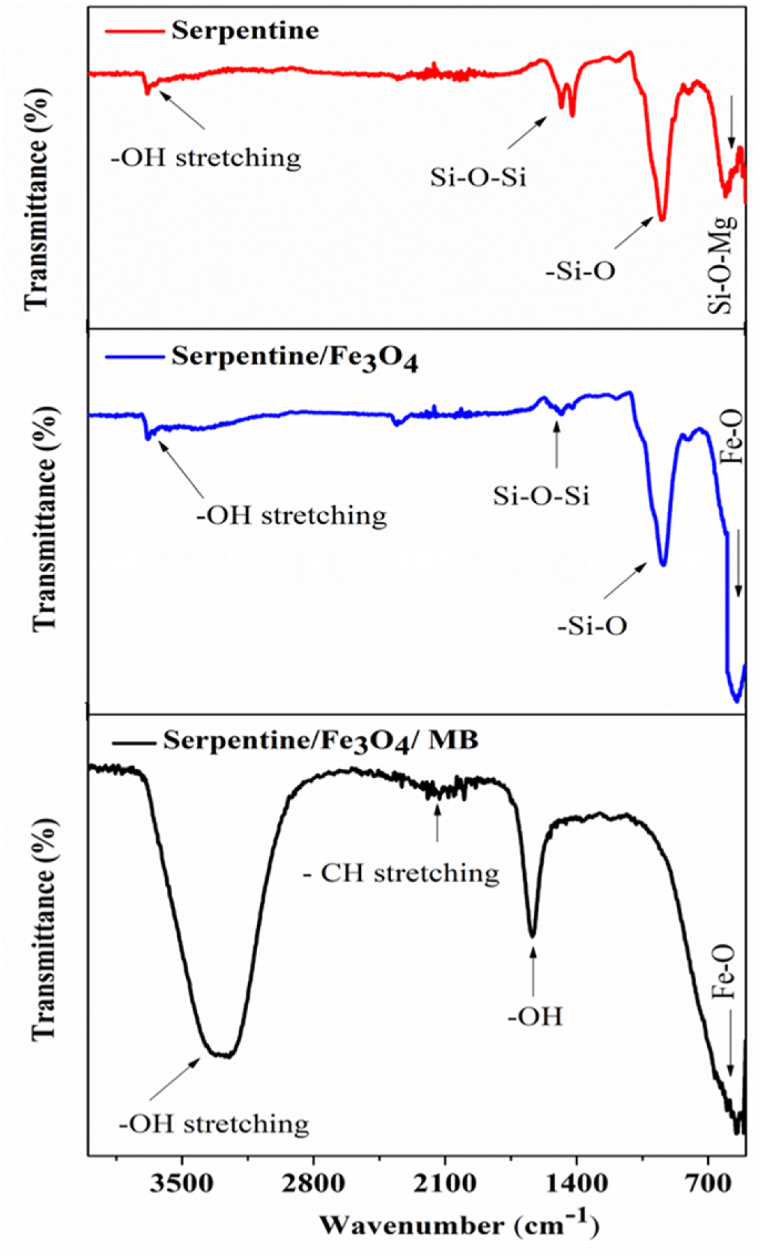
FTIR spectra of serpentine, Serpentine/Fe_3_O_4_ nanocomposite, and serpentine/Fe_3_O_4_/MB adsorbent.

Fig. 4 illustrates the comparison of FTIR spectra for the serpentine and serpentine/Fe_3_O_4_ nanocomposite. Serpentines are an assemblage of phyllosilicate minerals that possess hydroxyl groups (-OH) on their surface. These hydroxyl groupings can be employed to coordinate Fe metal ions. When Fe_3_O_4_ nanoparticles contact serpentine, the Fe ions present on the surface of nanoparticle can co-ordinate with the hydroxyl groups existing on the surface of serpentine [2]. For serpentine, serpentine/Fe_3_O_4_ nanocomposite, and serpentine/Fe_3_O_4_/MB adsorbent the attributed peak at 3689-3674 cm^−1^ or 1640 cm^−1^, 2126 cm^−1^, 1482-1489 cm^−1^, 934-939 cm^−1^, and 601-609 cm^−1^ due to the structural hydroxyl moiety of stretching vibration of Mg-OH and adsorbed water on the surface, C-H, Si-O-Si, Si-O_4_, Si-O-Mg bands of serpentine similar to bands mentioned for talc [8,[29], [30], [31], [32]]. The absorption band at 592-540 cm⁻^1^ corresponds to the Fe–O bond in Fe_3_O_4._ Hence, the FTIR analysis allows us to conclude that the Fe_3_O_4_ nanoparticles with a serpentine coating show distinctive absorption bands that match the Fe_3_O_4_ core and the serpentine covering.

### 3.4 Surface area analysis

**Fig. 5:**
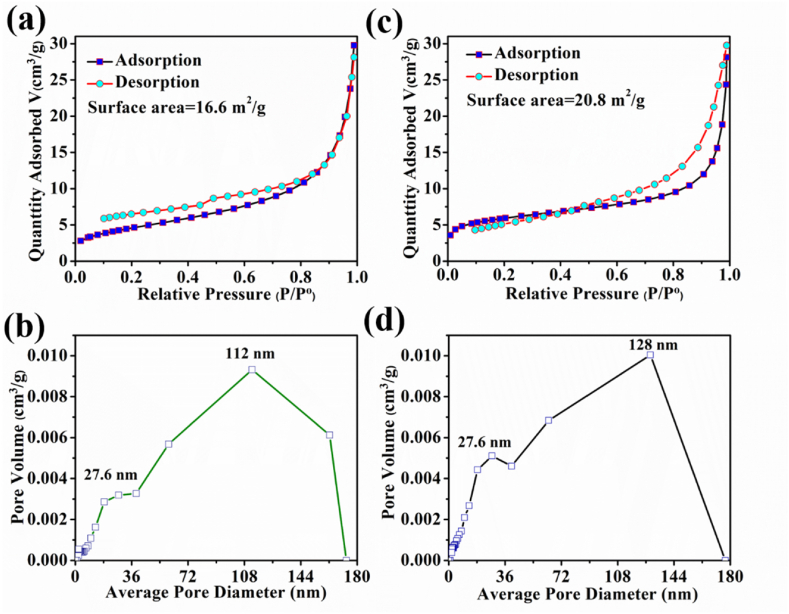
(a) Type-IV isotherm with a wide hysteresis loop of the serpentine (b) the pore size distribution of the serpentine (c) Type-IV isotherm with a wide hysteresis loop of the serpentine/Fe_3_O_4_ (d) the pore size distribution of the serpentine/Fe_3_O_4_ nanocomposite.

Fig. 5 (a) shows the N_2_ adsorption-desorption plot of serpentine/Fe_3_O_4_ nanocomposite and its pore size distribution. This allows the prediction that synthetic nanocomposite materials contain both macropores and mesopores. The pore size distribution obtained from the BJH analysis, the BJH curve shows narrow and intense peaks in the pore size distribution curve. The hole size distribution curve shows that they appear in a range of 1–150 nm. The specific surface area of serpentine is found to be 16.6 m^2^/g which is much smaller than that of the nanocomposite materials, which reaches 20.8 m^2^/g to enhance the active sites for adsorption [[33], [34], [35]]. Similar kinds of results were reported in the previous work. The highest portion of pore size obtained in nanocomposite was 128 nm, which confirms the detection of mesoporosity in the NPs. A very small number of macrospores (<50 nm) were also detected. The 20.8 m^2^/g surface area of this nanocomposite is moderate compared to similar magnetic nanocomposites such as Fe_3_O_4_/talc and Fe_3_O_4_/reduced graphene which have surface areas ranging from 30 to 150 m^2^/g [36,37]. The serpentine/Fe_3_O_4_ composite, while having surface area not as high as these advanced nanocomposites, still offers a good balance between surface area and cost-effectiveness. Moreover, it allows for better adsorption performance compared to pure serpentine. This improvement enables the nanocomposite to achieve a 98 % removal efficiency for methylene blue.

### 3.5 Magnetic properties

**Fig. 6:**
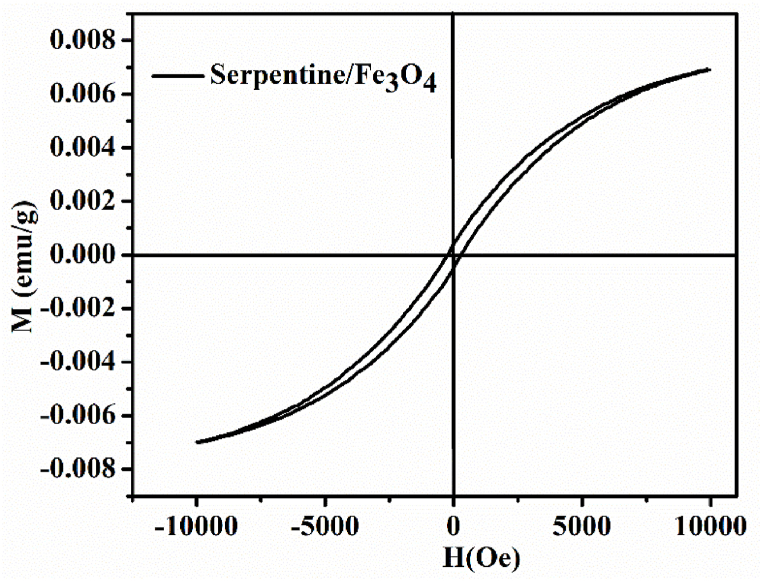
M − H Curve for serpentine/Fe_3_O_4_ nanocomposite.

The magnetic properties of the nanocomposite were analyzed by the vibrating sample magnetometer (VSM) at room temperature. The results of magnetic saturation of serpentine/Fe_3_O_4_ nanocomposite were 0.534 emu/g as shown in Fig. 6. Fei et al. reported the magnetic properties of polymer modified magnetic nanoparticles for the removal of cationic dyes using VSM [38]. Similarly, Li et al. investigated the saturation magnetization (13.3 emu/g) of magnetic guar gum-grafted carbon nanotubes for separating methylene blue from an aqueous solution [32]. The magnetization was decreased with the addition of serpentine due to large amount of non-magnetic serpentine. The reduced saturation magnetization of serpentine/Fe_3_O_4_ nanocomposite observed that the talc layers act as magnetic insulation bounded to nanoparticles [29]. The incorporated magnetic nanoparticles enhance the overall surface area available for dye adsorption. The magnetic adsorbent can then be separated from wastewater using an external magnetic field [7].

### 3.6 Effect of concentration, contact time, and adsorbent dose on methylene blue

**Fig. 7:**
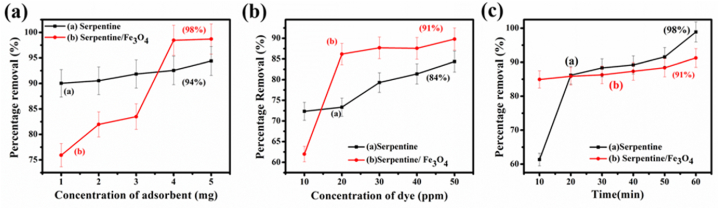
Factor affecting the percentage removal of MB (a) Concentration of adsorbent (b) Dye concentration (c) Agitation time.

The adsorbent dose resulted in 98 % removal of methylene blue in the case of the serpentine/Fe_3_O_4_ nanocomposite while it was 94 % in the case of serpentine. The increased adsorbent concentration leads to optimal adsorption efficiency at a 5 mg dose, as shown in Fig. 7(a). This is due to the large surface area and more accessible adsorption sites of the serpentine/Fe_3_O_4_ nanocomposite [2]. Increasing dye concentration leads to dye removal of 91 % for serpentine/Fe_3_O_4_ nanocomposites and 84 % for serpentine. This is due to the extended duration of interaction between the active site and the adsorbent/adsorbate, which leads to saturation and a decrease in adsorption capability, as illustrated in Fig. 7(b). Initially the removal of the MB abruptly increases that shows that at the time of contact between MB and composites there is more active site available that leads to continue regular pattern of removal in case of serpentine/Fe_3_O_4._ MB removal is carried out with agitation time, resulting in approximately 91 % removal for serpentine/Fe_3_O_4_ and 98 % for serpentine as shown in Fig. 7(c). The percentage removal increases with the increase in agitation time due to the gradual mass transfer from the bulk region to the adsorbent surface. In case of serpentine the adsorption increases with time because it is layered structure which increases the adsorption capacity and then shows the smaller increases after equal interval of time while the serpentine/Fe_3_O_4_ shows the regular pattern of dye removal. The better adsorption capability of serpentine/Fe_3_O_4_ nanocomposite is due to enhanced surface area as compared to serpentine as shown in Fig. 5. The addition of Fe_3_O_4,_ increases the composites magnetic properties which make separation of adsorbent easier and Fe_3_O_4_ mixed-valence state (Fe^2+^ and Fe^3+^) aid dye binding through redox interactions or electron transfer which facilitate dye molecule interaction (π-π∗ interaction or exposure of aromatic ring at metal side) between the nanocomposites and MB dye. The porous nature of the serpentine and the interstitial spaces between the serpentine and the Fe_3_O_4_ also improve the diffusion of the dye particles. The fundamental principle for the greater adsorption of the serpentine is a synergic effect, strong chemical interaction, and availability of active site between the serpentine and Fe_3_O_4_, as well as with MB dye.

However, various factors such as the saturation of the nanocomposites, aggregation, competitive adsorption, and desorption cause a decrease in the removal percentage after some time because Fe_3_O_4_ causes the saturation of the active sites (unavailability of the active sites) and the aggregation of the nanocomposite (the magnetic property leads to a lower surface area of the nanocomposites). It also creates a desorption ability that causes the nanocomposite ions to return to the solution. In contrast, serpentine itself is more stable and consistent in solution with gradually attaining equilibrium [[39], [40], [41]].

### 3.7 Adsorption isotherm modelling

**Fig. 8:**
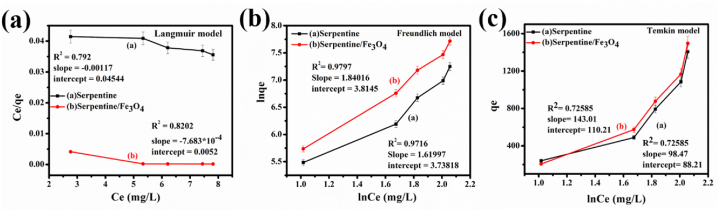
Various isotherm models (a) Langmuir and (b) Freundlich (c) Temkin model.

Langmuir, Freundlich, and Temkin isotherms were implemented to design a suitable adsorbent structure and optimize the adsorption process [42,43]. To determine the adsorption efficiency of serpentine and serpentine/Fe_3_O_4_ nanocomposite for the removal of MB, mathematical models were developed [2,44]. We used the Langmuir model to compute the adsorption onto the outer surface of materials. The adsorption data were fitted to the Langmuir model and the resulting Fig. 8 indicates that the R^2^ values for serpentine and serpentine/Fe_3_O_4_ nanocomposite were 0.792 and 0.8202, respectively. This formula was then applied to determine various adsorption characteristics in Equation (2).

Eq.2 $$\frac{C_{e}}{q_{e}}=\frac{C_{e}}{q_{m}}+\frac{1}{q_{m}b}$$

C_e_ represents the metal ion concentration at equilibrium on the nanocomposite surface, and q_e_ represents the organic pollutant mass at equilibrium. The maximum adsorption capacity of the serpentine/Fe_3_O_4_ nanocomposite is given by q_m_, which is a key parameter in the Langmuir model. The linear form of the Langmuir model allows us to determine q_m_ by analyzing the graph obtained by plotting C_e_/q_e_ against C_e_. The q_m_ value is obtained from the slope of the graph and then the intercept provides ‘b’ value, and the additional parameter, R_L_ (often known as separation factors), can be calculated using the following formula (equation (3)).

Eq. 3 $$R_{L}=\frac{1}{1+bC_{o}}$$

C_o_ represents the initial concentration of metal ions in the solution, while b stands for the Langmuir constant. The R_L_ factor ranges from 0 to 1 with higher R_L_ values indicating a more advantageous separation factor for MB. The q_m_ values for MB are about 854.70 and 1301 (mg/g), respectively, the R_L_ value is 0.0901 for serpentine and 0.0805 for serpentine/Fe_3_O_4_ nanocomposite. Serpentine shows Langmuir constant (b) of about 0.025744 (L/mg), whereas 1.3715 (L/mg) for serpentine/Fe_3_O_4_ nanocomposite. The R^2^ values demonstrate that the Langmuir model is suitable for describing the adsorption of organic pollutants on the surface of serpentine and serpentine/Fe_3_O_4_ nanocomposite as shown in Fig. 8(a). Freundlich isotherms were used to determine the adsorption efficiency of nanocomposites by using their linear form, equation (4).

Eq. 4 $$\ln\;q_{e}=\frac{1}{n}\ln\;C_{e}+\ln\;k_{f}$$

The values of n and k_f_ are the Freundlich constants determined by adsorption capacity and intensity by the graph constructed between ln q_e_ and ln C_e_ as shown in Fig. 8 (b). The value of n is smaller than 1 which means the chemical adsorption occurs in both serpentine and serpentine/Fe_3_O_4_ nanocomposite as shown in Table 1, along with R^2^ values [44]. The uptake of MB occurs at the heterogeneous active sites that build several layers (multilayers) onto the magnetic serpentine surface, according to the Freundlich equation. The development of monolayers is controlled by electrostatic interactions, and some dye molecules can be adsorbed by π-π interactions [45].

**Table 1:** Adsorption isotherm*s* constant for the adsorption of MB onto serpentine and serpentine/Fe_3_O_4_ nanocomposite.

Model	Parameters	Serpentine	Serpentine/Fe_3_O_4_
Langmuir model	q_m_ (mg/g)	854.70	1301
	k_L_ (L/mg)	0.02574	1.3715
	R^2^	0.7901	0.8202
	R_L_	0.0901	0.0895
Freundlich model	k_f_ (mg/g)	42.024	45.356
	n	0.6173	0.5434
	R^2^	0.9716	0.97971
Temkin model	B_T_ (J/mol)	2.5159	2.241
	R^2^	0.72585	0.7258501

In the case of serpentine, the Langmuir model is most suitable while in the case of serpentine/Fe_3_O_4_ nanocomposite, both Temkin and Freundlich models are suitably elaborated by R^2^ value as shown in Fig. 8(b) and (c). We prioritize the isotherm model by considering the R^2^ factor to ensure that the sequence holds for each organic pollutant. Equilibrium description is carried out by using the Temkin isotherm model where the time adsorption molecules' energy decreases gradually due to adsorbent/adsorbate interaction. The linear form of the Temkin model is given by Equation (5).

Eq. 5 $$q_{e}=K_{T}\;{\text{lnC}}_{e}+B_{T}$$

The values of B_T_ and K_T_ which are Temkin constants, were determined using the intercept and slope of q_e_ vs. ln (C_e_), as indicated in Table 1. It was shown that the q_e_ was a linear function of ln (C_e_), with R^2^ values of 0.72585 for serpentine/Fe_3_O_4_ nanocomposite produced by the co-precipitation technique and 0.7258 for the value of serpentine, respectively as shown in Fig. 8 (c). The Langmuir model's theoretical q_m_ does not accurately reflect the real adsorption capacity due to the low R^2^ value. The low value (0.8202) indicates a weak correlation between experimental data and the theoretical Langmuir model, making the calculated R_m_ unreliable. The higher R^2^ value for the Freundlich model (0.979) suggests that adsorption follows a multilayer mechanism with heterogeneous surface energies, which the Langmuir model cannot capture.

The adsorption efficiency for removing MB dye from the aqueous phase using various previously reported adsorbents is summarized in Table 2.

**Table 2:** Comparison of the maximum adsorption capacities of the serpentine/Fe_3_O_4_ nanocomposite for MB dye removal with various reported adsorbents.

Adsorbent	q_max_ (mg/g)	Dye Concentration (mg/L)	Temperature (°C)	Contact Time (min)	References
Polymer-modified using magnetic nanoparticles	142.90	50	30	120	[38]
Silver nanoparticles with grafted polymer of guar gum/acrylic acid	833.33	100	25	90	[46]
Magnetic guar gum grafted with carbon nanotubes	61.92	20	30	60	[32]
Reduced graphene oxide with silver nanoparticles	970	50	25	150	[47]
Xylan/poly (acrylic acid) with magnetic nanocomposite	438.60	25	25	120	[48]
Natural serpentine/Fe_3_O_4_ nanocomposite	392	20	25	60	Present study

### 3.8 Adsorption kinetics

Among the kinetic models used to clarify the mechanism of MB adsorption by serpentine and serpentine/Fe_3_O_4_ nanocomposites were the pseudo-1st-order, pseudo-2nd-order, intra-particles diffusion model, and Elovich model. Because there were multiple sites accessible for MB adsorption, the serpentine/Fe_3_O_4_ nanocomposites performed exceptionally well during the early adsorption stage. The adsorption rate constant and regulating mechanism were calculated using the pseudo-first-order and pseudo-second-order kinetics models. The pseudo-1st order (Eq. (6)) and second-order kinetic (Eq. (7)) models have the following linear forms.

Eq. 6 $$\ln\left(q_{e}-q_{t}\right)=k_{1}t+\ln\;q_{e}$$

**Fig. 9:**
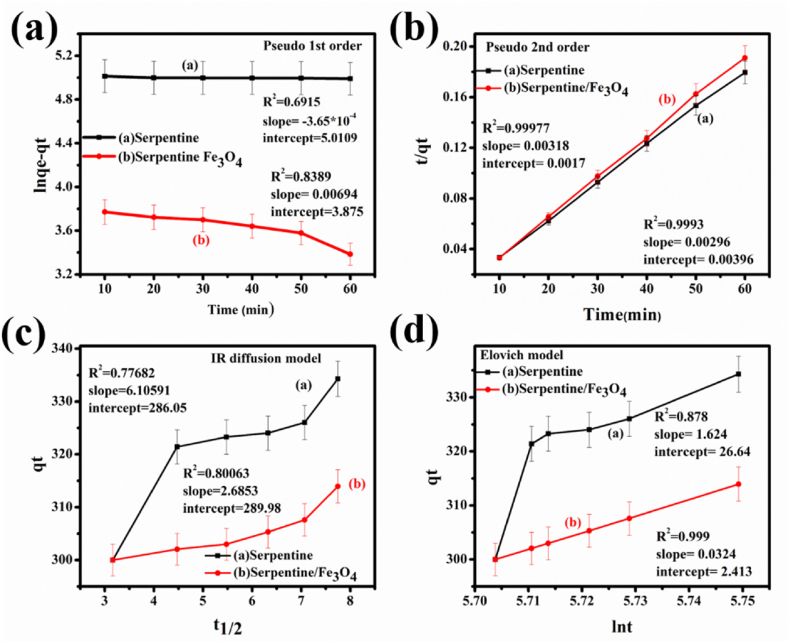
Adsorption kinetics (a) Pseudo 1st order, (b) Pseudo 2nd order, (c) IR model, (d) Elovich model.

Eq. 7 $$\frac{t}{q_{t}}=\frac{1}{k_{2}q_{e}^{2}}+\frac{t}{q_{e}}$$

where q_e_ is the mass of the MB adsorbed per unit mass at equilibrium and q_t_ is the mass of organic pollutants (MB) adsorbed at the surface of serpentine and serpentine/Fe_3_O_4_ nanocomposites at any given point. The slope of plot of ln (q_e_-q_t_) vs. time shown in Fig. 9(a) yields the pseudo-first-order rate constants for serpentine and serpentine/Fe_3_O_4_, which is −3.6 × 10^−4^, and 0.00694, respectively. The R^2^ values for serpentine (0.6915) and serpentine/Fe_3_O_4_ nanocomposites (0.8389) are displayed in Table 3. Based on the slope and intercept values of the plot of t/q_t_ vs. time as shown in Fig. 9(b), equation (7) was utilized to get the pseudo-second-order rate constant (k_2_) for MB. The k_2_ values for MB with two distinct sample types are - 2.21 × 10^−3^ for serpentine and −5.94 × 10^−3^ for serpentine/Fe_3_O_4_ are shown in Table 3. Serpentine/Fe_3_O_4_ nanocomposites are adsorbing organic contaminants by pseudo-second-order kinetics, as indicated by the R^2^ value which is almost equal to 1. The intra-particles diffusion model helps in the refined adsorption mechanism and this model rate constant, K_d_, is derived by plotting the graph of q_t_ vs. t ^½^ (Fig. 9 (c)), and the boundary thickness [C] is defined by the linear form of equation (8).

Eq 8 $$q_{t}=k_{d}t^{1/2}+C$$

**Table 3:** Adsorption kinetics models for serpentine and serpentine/Fe_3_O_4_ nanocomposites.

Kinetics	Parameters	Serpentine	Serpentine/Fe_3_O_4_
Pseudo 1st order	K_1_ (min^−1^)	−3.6 × 10^−4^	0.00694
	R^2^	0.6915	0.839
Pseudo 2nd order	K_2_ (mg/g/min)	2.21 × 10^−3^	5.94 × 10^−3^
	R^2^	0.9993	0.9997
Intra-particle diffusion model	k_d_ (mg/g/min^1/2^)	6.10591	2.685
	R^2^	0.7768	0.8003
Elovich model	α (mg/g/min)	111.25	37242.28
	β (g/mg)	574.4	0.003
	R^2^	0.878	0.999

Because MB ions were traveling instantly in the direction of the nanocomposite surface, the value of q_t_ increased steadily with t^1/2^. Due to the energetically heterogeneous structure, the Elovich model supports second-order kinetics. Plotting the graph between lnt and time in the Elovich model (Fig. 9 (d)) allows us to calculate the values of α and β using Equation. 9

Eq 9 $$q_{t}=\frac{1}{\beta }\ln\;\left(\alpha \beta \right)+\frac{1}{\beta }lnt$$

where the β value is 0.003 g/mg and α value is 37242.281 for serpentine/Fe_3_O_4_ nanocomposites, and 574.4 g/mg, and 111.25 for serpentine respectively. The value of R^2^ given in Table 3 indicated the heterogeneous nature of the serpentine/Fe_3_O_4_ nanocomposites with better results as compared to serpentine [44]. In the Elovich model the β value is related to the desorption capacity of the nanocomposites so that by comparing the value we can check the desorption capacity of these nanocomposites.

## 4 Advantages of serpentine, and serpentine/Fe_3_O_4_ nanocomposites

Table 2 shows adsorption capacity of other reported adsorbent in comparison to serpentine and serpentine/Fe_3_O_4_ nanocomposites ranging from 800 to 1300 mg/g. The adsorption capacity is influenced by surface area and pore size which is 16.6 m^2^/g and 112 nm for serpentine and 20.6 m^2^/g and 128 nm for serpentine/Fe_3_O_4_. FTIR analysis confirms the presence of the oxygen-containing functional groups. The large surface area, pore size, and functional group lead to greater adsorption ability. Fe_3_O_4_ when combined with the serpentine increases the magnetic property results in easy separation of adsorption from the water for further reusability. The layered structure of the serpentine with Fe_3_O_4_ presence increases the adsorption sites for the removal of the MB dye as shown in the Freundlich adsorption isotherm (Linear in Fig. 8(b), and non-linear in supplementary file). These nanocomposites also favor selective adsorption because the MB is a positive charge dye and the Fe_3_O_4_ has a negative charge that leads to the removal of the dye. The main advantages of the nanocomposites are reusability capability, low cost, stability (shown in the PL spectra, and FTIR spectra), and surface interaction.

Hence, the adsorption of MB by serpentine/Fe_3_O_4_. ​ is mainly governed by surface area, functional groups (hydroxyl groups), magnetic properties, and electrostatic interactions, with the synergistic effect between serpentine and serpentine/Fe_3_O_4_. playing a key role in enhancing adsorption efficiency.

## 5 Conclusions

In summary, the study demonstrated the successful synthesis of serpentine/Fe_3_O_4_ nanocomposites using a straightforward co-precipitation method, with serpentine sourced naturally from Skardu, Pakistan. The nanocomposites exhibited enhanced adsorption capabilities for MB dye, achieving a removal efficiency of 98 % under optimal conditions. This high performance is attributed to the synergistic combination of serpentine's layered structure and hydroxyl groups with the magnetic and adsorption properties of Fe_3_O_4_ nanoparticles. Various characterization techniques, including FTIR, UV, SEM, EDS, VSM, XRD, and surface area analysis provided the physical and chemical properties of the nanocomposite. The adsorption process followed the Freundlich isotherm model (R^2^ = 0.978) that indicates multilayer adsorption on a heterogeneous surface. The kinetics of the adsorption process fit well with the pseudo-second-order model (R^2^ = 0.9993) which suggest the chemisorption is the primary mechanism of dye removal. The nanocomposite's high surface area (20.8 m^2^/g) and magnetic properties (saturation magnetization of 0.534 emu/g) enabled effective dye adsorption and easy separation using an external magnetic field. The present work is novel in utilizing naturally abundant serpentine to develop cost-effective and environmentally friendly adsorbents with high adsorption capacity for wastewater treatment. However, challenges such as Fe_3_O_4_agglomeration, which reduces surface availability over prolonged use, were identified. Future work will explore advanced synthesis methods to overcome these limitations and evaluate the performance of the composite in removing a broader range of pollutants. The findings highlight the potential of serpentine/Fe_3_O_4_ nanocomposites as practical, scalable solutions for industrial wastewater treatment, contributing to sustainable water management practices.

## CRediT authorship contribution statement

**Amtul Basit:** Conceptualization, Investigation, Methodology, Resources, Validation, Writing – original draft. **Zahida Yaqoob:** Conceptualization, Data curation, Investigation, Methodology, Writing – original draft. **Aliya Zahid:** Conceptualization, Investigation, Methodology, Writing – review & editing. **Shaista Ali:** Investigation, Methodology, Writing – review & editing. **Beenish Shoukat:** Investigation, Methodology, Software, Writing – original draft. **Abdul Khaliq:** Investigation, Methodology, Resources, Software, Writing – review & editing. **Muhammad Tajammal Chughtai:** Investigation, Methodology, Writing – original draft, Writing – review & editing. **Rahila Batul:** Methodology, Software, Validation, Writing – review & editing. **Muhammad Atiq Ur Rehman:** Conceptualization, Methodology, Project administration, Resources, Supervision, Visualization, Writing – review & editing. **Syed Wilayat Husain:** Conceptualization, Investigation, Methodology, Project administration, Supervision, Writing – review & editing.

## Data availability statement

Data will be available upon request.

## Declaration of competing interest

The authors declare that they have no known competing financial interests or personal relationships that could have appeared to influence the work reported in this paper.
